# Radiotherapy can improve overall survival in patients with lymph-node positive, high-grade neuroendocrine cervical cancer: construction of two prognostic nomograms to predict treatment outcome

**DOI:** 10.3389/fonc.2024.1450382

**Published:** 2024-09-13

**Authors:** Siying Zhang, Qinke Li, Xiping Ouyang, Ya Tang, Ji Cui, Zhu Yang

**Affiliations:** ^1^ Department of Gynecology and Obstetrics, the Second Affiliated Hospital of Chongqing Medical University, Chongqing, China; ^2^ Department of Gynecology and Obstetrics, the First Affiliated Hospital of Chongqing Medical University, Chongqing, China

**Keywords:** high-grade neuroendocrine cervical cancer, survival prognosis, SEER database, radiotherapy, nomogram

## Abstract

**Background:**

To explore the beneficial subgroups after radiotherapy in high-grade neuroendocrine cervical cancer (HGNECC) and construct two survival prognosis models to quantify the efficacy of radiotherapy assessment.

**Methods:**

In this retrospective study, we included 592 eligible samples from the Surveillance, Epidemiology, and End Results (SEER) database and 56 patients with lymph-node positive HGNECC from Chongqing Medical University. Cox regression analysis was used to identify independent survival prognosis risk factors for HGNECC patients. Propensity score matching (PSM) was employed as it balances the baseline differences among grouping methods. Kaplan–Meier (K-M) curves were used to analyze survival differences among different groups. Two survival prediction nomograms were constructed separately (using the "rms" package in R software) based on whether radiotherapy was administered. The stability and accuracy of these models were assessed using receiver operating characteristic (ROC) curves and calibration curves in both the training and validation datasets. P<0.05 was considered to indicate statistically significant differences.

**Results:**

Age, Federation of Gynecology and Obstetrics (FIGO)-stage, and treatment methods (surgery vs. chemotherapy) were independent risk factors that affected survival prognosis (P<0.05). Radiotherapy showed adverse effects on survival in patients with early tumor staging, lymph-node negative status, and absence of distant metastasis (all P<0.05). The lymph-node positive group had a beneficial response to radiotherapy (P<0.05), and patients with metastasis in the radiotherapy group showed a survival protection trend (P=0.069).

**Conclusion:**

In HGNECC, patients with lymph-node positive status can benefit from radiotherapy in terms of survival outcomes. We constructed two survival prediction models based on whether radiotherapy was administered, thereby offering a more scientifically guided approach to clinical treatment planning by quantifying the radiotherapy efficacy.

## 1 Introduction

Neuroendocrine cervical cancer (NECC) is a rare malignant tumor originating from the cervix, and accounts for approximately 1–1.5% of all cervical malignancies (1). Saavedra et al. first described NECC in 1972 and then categorized it into four main types in 1976 (2). The current WHO classification further categorizes NECC into low-grade with relatively favorable prognosis (carcinoid and atypical carcinoid) and high-grade with worse prognosis (small-cell neuroendocrine carcinoma and large cell neuroendocrine carcinoma, accounting for 80% and 12%, respectively) (1). Given its rarity, the knowledge and treatment of NECC mainly rely on case reports or small sample studies. To our knowledge, there is still no established guideline for managing NECC, which presents a significant challenge in understanding and treating this tumor.

Currently, the treatment approach for NECC is similar to that of cervical adenocarcinoma (AC) or squamous cell carcinoma (SCC). Radical hysterectomy followed by adjuvant radiotherapy or chemotherapy remains the main treatment approach in early-stage NECC. In advanced-stage NECC, a combination of radiotherapy, chemotherapy, and personalized comprehensive treatment is preferred (3). AC/SCC predominantly spreads locally, with a lower incidence of distant metastasis. Radiotherapy, as a key adjuvant treatment, plays a crucial role in suppressing local recurrence, metastasis, thereby enhancing both progression-free survival (PFS), overall-survival (OS), and contributing to favorable long-term survival outcomes. In contrast, NECC has a lower 5-year survival rate of approximately 36% and a median survival period ranging from 22 to 25 months. For the more aggressive small cell neuroendocrine carcinoma of the cervix, the 5-year survival rate is typically less than 2 years (4).

Because NECC is characterized by its highly metastatic nature, surgery in the early stage and adjuvant chemotherapy in all stages are all universally accepted as effective treatment options, regardless of its subtype (5–8).However the efficacy of radiotherapy remains uncertain (6, 8–11). Some studies suggest that combined radiotherapy does not significantly improve survival outcomes but certain subgroups may benefit from this approach. Factors such as age, staging, and lymph node status may influence radiotherapy effectiveness (12–16). Owing to the complexity of individual variables and limited sample sizes, no study has yet comprehensively described the treatment effects of radiotherapy on NECC or quantified the association between prognostic risk factors affecting radiotherapy benefits and survival outcomes. NECC primarily affects young-to-middle-aged women, emphasizing quality of life and survival benefits (17). Hence, further exploration using high-quality, large-sample data are needed for tailored treatment strategies in NECC patients. Our study aims to explore factors affecting radiotherapy outcomes, construct survival prediction models for both radiotherapy and non-radiotherapy groups, and validate these models using clinical data. Successful development of these models will help identify patients who can benefit from radiotherapy, tailor treatment plans, and improve the OS rates.

## 2 Materials and methods

### 2.1 Data resource

This was a retrospective clinical cohort study, some of the data were obtained through the SEER*Stat software (V8.3.6) from the SEER public database (https://seer.cancer.gov/). The SEER database, established by the National Cancer Institute in 1973, collects comprehensive cancer data from various regions and subpopulations across the United States. It currently includes data from 18 registration centers, covering a wide range of tumor types. Meanwhile, another Patient data collected from the First and the Second Affiliated Hospital of Chongqing Medical University for the past decade (January 2010 to December 2020).

### 2.2 Patients selection

This study screened patient data from the Surveillance, Epidemiology, and End Results (SEER) database. Data of all patients in 18 registration centers in U.S from 1988 to 2019 were retrieved, initially including 1342 cases of confirmed cervical neuroendocrine carcinoma. The patients selection process is as follows: (1) the primary sites selected were C53.0-Endocervix, C53.1-Exocervix, C53.8-Overlapping lesion of cervix uteri, and C53.9-Cervix uteri; (2) according to the International Classification of Diseases for Oncology, 3rd Edition (ICD-O-3), the initial codes proposed for inclusion were 8013/3 (large cell neuroendocrine carcinoma), 8041/3 (small cell neuroendocrine carcinoma), 8045/3 (mixed small cell carcinoma).

Additionally, 66 cases from Chongqing Medical University's affiliated hospitals (2010-2020) were collected. All included patient data were confirmed by the pathology department of our hospital, with a clear pathological diagnosis of cervical neuroendocrine tumors. Both groups have complete follow-up and known survival status.

**Figure 1:**
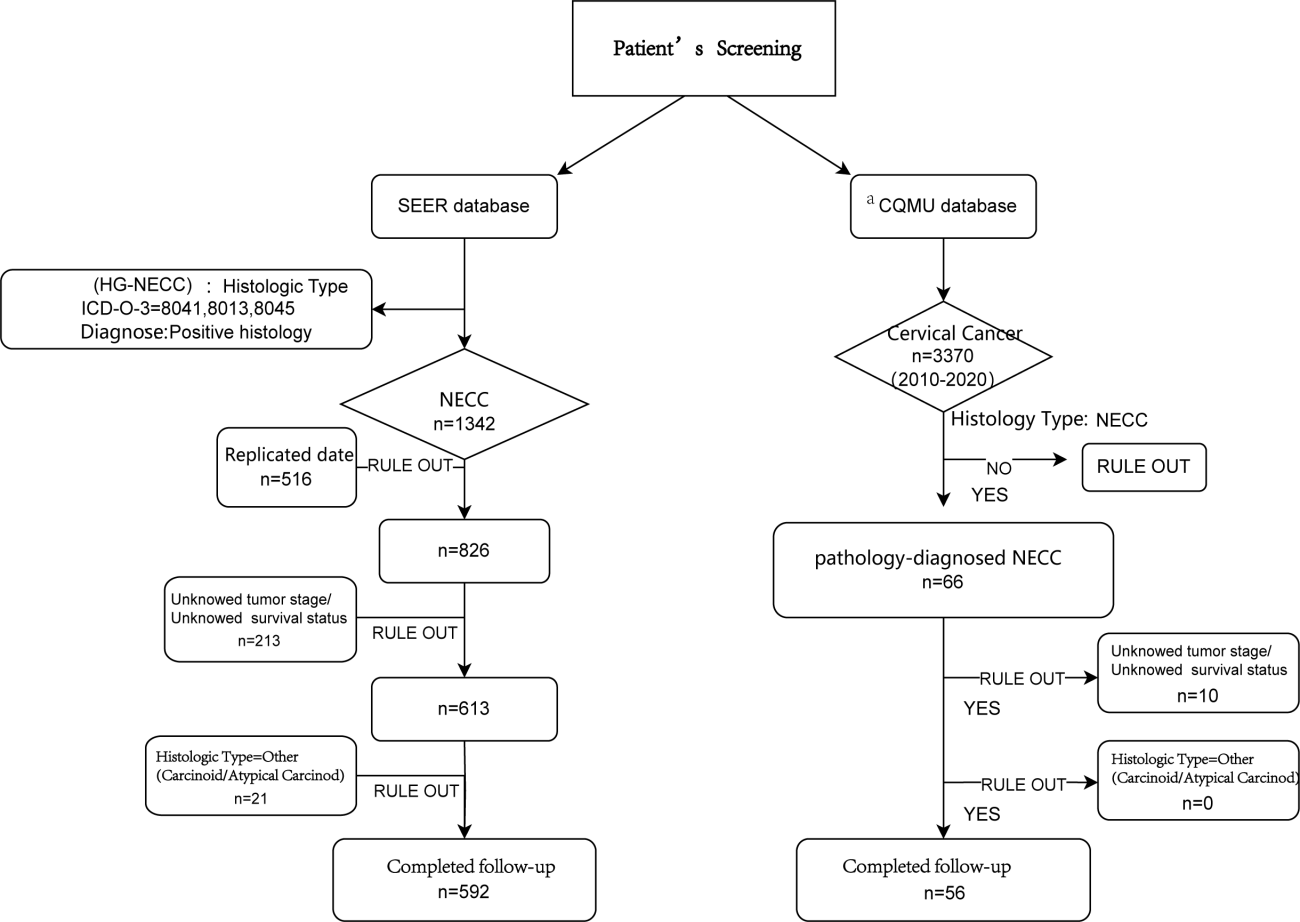
The flowchart of enrolled patients screening. ^a^CQMU, Chongqing Medical University. Here refers to the First and Second Affiliated Hospitals specifically.

The exclusion criteria were secondary tumors, duplicate data, and insufficient records. Notably, owing to the limited sample size of low-grade NECCs (<30 cases), patients classified histologically as "other" were excluded. This study focused only on high-grade neuroendocrine carcinomas (HGNECC). The data screening process is shown in **Figure 1**.

### 2.3 Variables definition

This study examines patient baseline characteristics and survival status, ensuring data availability in both the SEER database and our hospital's records. Baseline data include demographic factors: Patient ID, Year of diagnosis, Age at diagnosis; Tumor features; Historical stage (AJCC 7th edition), Grade, Primary site, TNM staging, FIGO staging; Treatment details: Surgery, Radiotherapy, Chemotherapy; Follow-up: Survival time, Survival status. Overall Survival (OS) is defined as the time from NECC diagnosis to the last follow-up, with causes of death not limited to NECC.

### 2.4 Statistical analysis

Continuous variables are described as mean±standard deviation (SD) or median with interquartile range (IQR) values, depending on whether their distribution was normal or non-normal. Categorical variables are shown as numbers and percentages for each group. We used Cox proportional hazards regression analysis to identify factors affecting survival prognosis. Considering variations in the effectiveness of radiotherapy, we analyzed subgroup survival differences across different grouping methods. Propensity score matching (PSM) was employed to balance baseline data among the various groups. Kaplan–Meier (KM) survival curves were plotted to compare survival outcomes. Participants were divided into radiotherapy and non-radiotherapy groups, and two nomogram models were constructed using the "rms" package in R. Receiver operating characteristic (ROC) and calibration curves were used to visually assess the predictive performance of the models. All statistical analyses were performed using R software (version 3.6.3). P<0.05 was considered to indicate statistically significant differences.

## 3 Results

### 3.1 Clinical characteristics

Analysis of baseline data (**Table 1**) from both the SEER database (591 cases) and our hospital database (56 cases) revealed the following: 1) The prognosis for patients in both databases was not optimistic, especially in our hospital database (mean OS: 37.7 months, median survival time: 35.2 months). Over 50% patients had a status of death in the two databases (69.9% vs. 58.9%); 2) age<60 years was the main affected group (73.4% vs. 94.7%), and small cell neuroendocrine carcinoma was the predominant pathological type in HGNECC (89.2% vs. 48.2%); and 3) the majority of patients in both databases underwent surgery and chemotherapy, with a smaller proportion opting for radiotherapy (27.7% vs 35.7%).

**Table 1:** Baseline data of all HGNECC patients.

	SEER	CQMU^*^
	(N=591)	(N=56)
Survival time		
Mean (SD)	49.4 (75.2)	37.7 (25.5)
Median [Min, Max]	16.0 [0, 383]	35.2 [11.2, 120]
Survival Status		
Alive	184 (31.1%)	23 (41.1%)
Death	407 (68.9%)	33 (58.9%)
Age		
<40	194 (32.8%)	16 (28.6%)
40-60	240 (40.6%)	37 (66.1%)
>60	157 (26.6%)	3 (5.4%)
Primary Site		
Cervix uteri	503 (85.1%)	38 (67.9%)
Endo-cervix/Other	88 (14.9%)	18 (32.1%)
Histologic Type		
Large	64 (10.8%)	29 (51.8%)
Small	527 (89.2%)	27 (48.2%)
Grade		
Moderately/well differentiated	6 (1.0%)	0 (0.0%)
Poorly/Un- differentiated	340 (57.5%)	6 (10.7%)
Unknown	245 (41.5%)	50 (89.3%)
FIGO Stage		
I	156 (26.4%)	23 (41.1%)
II	45 (7.6%)	18 (32.1%)
III	163 (27.6%)	12 (21.4%)
IV	227 (38.4%)	3 (5.4%)
T		
T1	237 (40.1%)	23 (41.1%)
T2	130 (22.0%)	18 (32.1%)
T3	126 (21.3%)	11 (19.6%)
T4	30 (5.1%)	4 (7.1%)
TX/Unknown	68 (11.5%)	0 (0%)
N		
N0	269 (45.5%)	43 (76.8%)
N1	259 (43.8%)	13 (23.2%)
NX	63 (10.7%)	0 (0%)
M		
M0	373 (63.1%)	55 (98.2%)
M1	218 (36.9%)	1 (1.8%)
Surgery		
No	309 (52.3%)	5 (8.9%)
Yes	282 (47.7%)	51 (91.1%)
Chemotherapy		
No/Unknown	140 (23.7%)	1 (1.8%)
Yes	451 (76.3%)	55 (98.2%)
Radiation		
No	427 (72.3%)	36 (64.3%)
Yes	164 (27.7%)	20 (35.7%)

*Chongqing Medical University. Here refers to the First and Second Affiliated Hospitals specifically.

SD, standard deviation; OS, overall survival; HGNECC, high-grade neuroendocrine cervical cancer.

### 3.2 Risk factors for overall survival of HGNECC patients

Due to both T-stage and Federation of Gynecology and Obstetrics (FIGO)-stage being included in the baseline data of all patients from the SEER database, there may be potential overmatching in subsequent Cox analysis and PSM. As for another indicator of grade, 50% patients are classified as poorly differentiated, while the grade of the remaining 50% is unknown. Moreover, obtaining T stage and grade information from our database is challenging. Thus, we plan to exclude T-stage and tumor grade in the subsequent analysis. Cox regression analysis of another 10 subjects from the SEER database (591 cases) identified risk factors affecting OS (**Table 2**). Among them, age, FIGO-stage, treatment strategies (surgery vs. chemotherapy) emerged as independent risk factors affecting long-term survival (P<0.05). Surgical group showed a 50% reduction in the risk of death compared to non-surgical group (HR=0.488, P<0.05), while chemotherapy led to a 53% decrease in the mortality risk (HR=0.47, P<0.05). Although initial analysis indicated a 43% lower mortality risk with radiotherapy, further multi-variable Cox analysis revealed a 25% increase in mortality risk compared to non-radiotherapy treatment (HR=1.25, P=0.135), suggesting the potential adverse effect of HGNECC on OS rate.

**Table 2:** Cox hazards regression analysis of OS in patients (HGNECC) from SEER database.

Characteristics	Univariable model				Multivariable model			
	HR	HR.95L	HR.95H	P-value	HR	HR.95L	HR.95H	P-value
**Age**	2.12	1.696	2.65	<0.001				
<40	1	–	–	–				
40-60	1.668	1.306	2.131	<0.001	1.262	0.975	1.633	0.077
**>60**	**3.239**	**2.503**	4.192	<0.001	**2.105**	**1.587**	**2.79**	**<0.001**
Primary Site								
Cervix uteri	1	–	–	–				
Endo-cervix/Other	0.793	0.600	1.050	0.103				
Histologic Type								
Large								
Small	0.796	0.586	1.083	0.147				
FIGO_Stage								
I	1	–	–	–				
II	**1.722**	**1.111**	**2.670**	**0.015**	1.061	0.666	1.688	0.804
**III**	**2.304**	**1.709**	**3.106**	**<0.001**	**1.922**	**1.300**	**2.840**	**0.001**
**IV**	**5.475**	**4.129**	**7.258**	**<0.001**	**3.007**	**1.534**	**5.895**	**0.001**
N								
N0	1	–	–	–				
N1	**1.988**	**1.606**	**2.461**	**<0.001**	1.234	0.918	1.659	0.163
NX	**3.208**	**2.355**	**4.371**	**<0.001**	1.196	0.837	1.709	0.326
M								
M0	1	–	–	–				
M1	**3.321**	**2.711**	**4.068**	**<0.001**	1.273	0.716	2.266	0.411
Surgery								
No	1	–	–	–				
**Yes**	**0.38**	**0.31**	**0.465**	**<0.001**	**0.516**	**0.396**	**0.673**	**<0.001**
Chemotherapy								
No	1	–	–	–				
**Yes**	**0.59**	**0.48**	**0.741**	**<0.001**	**0.470**	**0.372**	**0.594**	**<0.001**
Radiotherapy								
No	1	–	–	–				
**Yes**	0.567	0.452	0.711	**<0.001**	1.255	0.936	1.683	0.128

HR, hazard ratio; OS, overall survival; CI, confidence interval. The bold values denote statistical significance at P < 0.05 level.

### 3.3 Discussing the efficacy of radiotherapy in HGNECC patients

**Figure 2:**
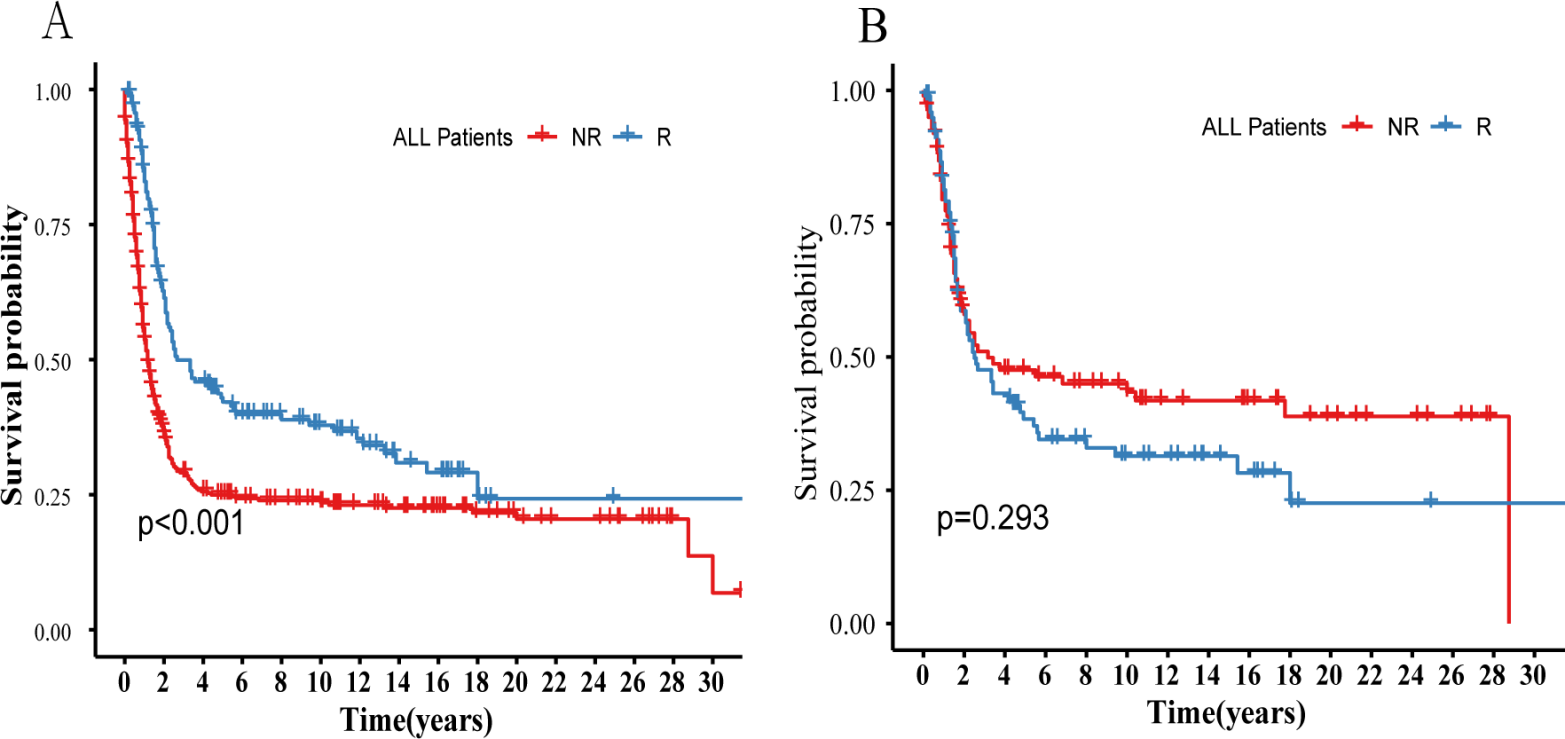
Compares the survival differences between the R and NR groups before and after PSM in all patients in the SEER database. **(A)** Survival probability before PSM. **(B)** Survival probability after PSM. R, Radiotherapy; NR, Non radiation therapy.

To better understand the benefit of radiotherapy, all subjects from the SEER database were grouped based on whether radiotherapy was administered. Balancing baseline data with PSM (**Supplementary Table S1**), KM curves then depicted survival differences between groups before and after PSM (**Figures 2A, B**). The results obtained align with the findings from the Cox regression analysis regarding the benefits of prior radiotherapy (**Table 2**). Despite no clear advantage in OS with radiotherapy after PSM (P>0.05), both Unix-variables Cox analysis and inter-group comparisons before PSM demonstrated significant survival benefits in the R-group (P<0.05). This suggests potential influencing factors affecting the benefit of radiotherapy. We analyzed all observed indicators included in the study, and subsequent analysis explored some specific indicators related to radiotherapy efficacy, grouping subjects by various criteria, and analyzing survival differences between the radiotherapy (R) and non-radiotherapy (NR) groups.

#### 3.3.1 Analysis of radiotherapy efficacy in different stages

**Figure 3:**
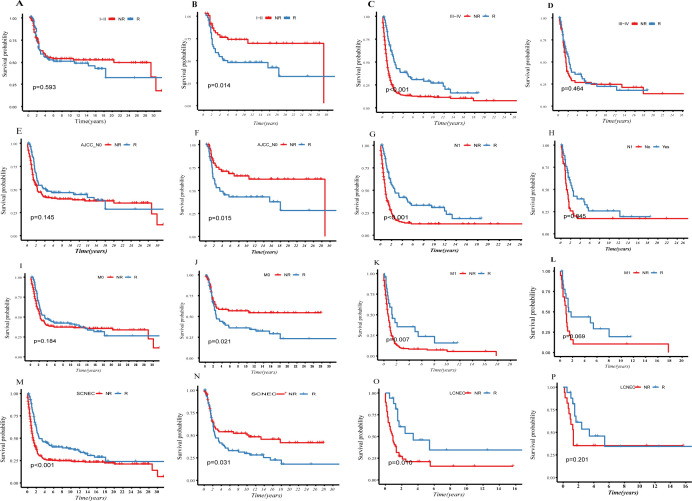
Compare the survival difference before and after PSM between the R- and NR-group in different indicators: **(A–D)**, grouping according to FIGO staging; **(E–H)**, grouping according to lymph nodes status; **(I–L)**, grouping according to whether distant metastasis; **(M–P)**, grouping according to histological difference.

We categorized patients into early-stage (stage I–II) and advanced-stage (stage III–IV) groups based on the FIGO-stage (**Figures 3A–D**). PSM was used to balance the baseline data (**Supplementary Table S2**). The KM curves depict survival differences between the R and NR groups. The addition of radiotherapy in early-stage patients had a statistically significant adverse-effect on survival prognosis (**Figures 3A, B**, P=0.014). Conversely, no significant survival differences were noted in advanced-stage patients (P>0.05, **Figures 3C, D**).

#### 3.3.2 Analysis of radiotherapy efficacy in different lymph-node status

We categorized patients from the SEER database into two groups (**Figures 3E–H**) based on lymph node status: N0 (negative) and N1 (positive). PSM was used to balance the baseline data (**Supplementary Table S3**). KM depicts survival differences between the R and NR groups. After PSM, radiotherapy had a statistically significant adverse effect on survival prognosis in the N0 group (P=0.015, **Figures 3E, F**), while it showed a clear survival protective effect in the N1 group (P=0.045, **Figures 3G, H**).

#### 3.3.3 Analysis of radiotherapy efficacy based on the presence and absence of distant metastasis

We categorized patients from the SEER database into two groups (**Figures 3I–L**): M0 (no distant metastasis) and M1 (distant metastasis). PSM was used to balance the baseline data (**Supplementary Table S4**). The KM curves depict survival differences between subgroups. After PSM, it was observed that radiotherapy was a clear risk factor in the M0 group (P=0.002, **Figures 3I, J**). Although radiotherapy did not reach statistical significance, it demonstrated a certain protective trend in the M1 group (P=0.069, **Figures 3K, L**).

#### 3.3.4 Analysis of radiotherapy efficacy based on histological differences

We categorized patients from the SEER database into two groups (**Figures 3M–P**): LCNEC and SCNEC. We balanced the baseline data using PSM (**Supplementary Table S5**). KM curves depict survival differences between subgroups. Radiotherapy was identified as a clear prognostic risk factor in SCNEC, which has poorer differentiation and higher malignancy than LCNEC (P=0.031, (**Figures 3M–P**).

Our analysis indicates that NECC tends to respond poorly as the malignancy progresses, and the local control benefits of radiotherapy diminish. We also observed the patients with SCNEC had worse OS than LCNEC after radiotherapy (P=0.031, **Figures 3O, P**). Additionally, in early-stage and lymph node-negative groups, those who did not receive radiotherapy showed significantly better OS (**Figures 3B, F**). However, there are patients who can benefit from radiotherapy: those with lymph-node positive status exhibited a clear survival advantage in the R-group (**Figure 3H**). Although our study did not show clear benefits in the M1 group, there was a protective trend in the R-group (**Figures 3K, L**).

### 3.4 Constructing survival prediction models based on radiotherapy efficacy

Because of the complexity of individual cases, determining whether to add radiation therapy can be challenging when both favorable and unfavorable factors are present. The nomogram, integrating multiple prognostic factors, offers a personalized assessment tool. Based on multi-variable Cox regression analysis, the nomogram combines independent prognostic indicators to calculate their respective weights in predicting survival outcomes.

Following this principle, we divided the SEER database population into two groups: the R and NR groups. Cox regression analysis was used to identify the factors influencing survival outcome in each group independently: We constructed nomograms for both groups, validating both models with data from the SEER database (internal validation group) and our hospital's system (external validation group). Through objective scoring, we quantified the population benefiting from radiation therapy.

#### 3.4.1 Construction and validation of nomogram for NR-group

We began by constructing the predictive model for the NR group. There were 427 samples without radiation therapy in the SEER database. Splitting them in a 7:3 ratio, the computer randomly divided them into a training group (299 patients) and an internal validation group (128 patients). Additionally, we considered 36 non-radiotherapy patients from our institution's database as the external validation group (**Supplementary Table S6**).

**Figure 4:**
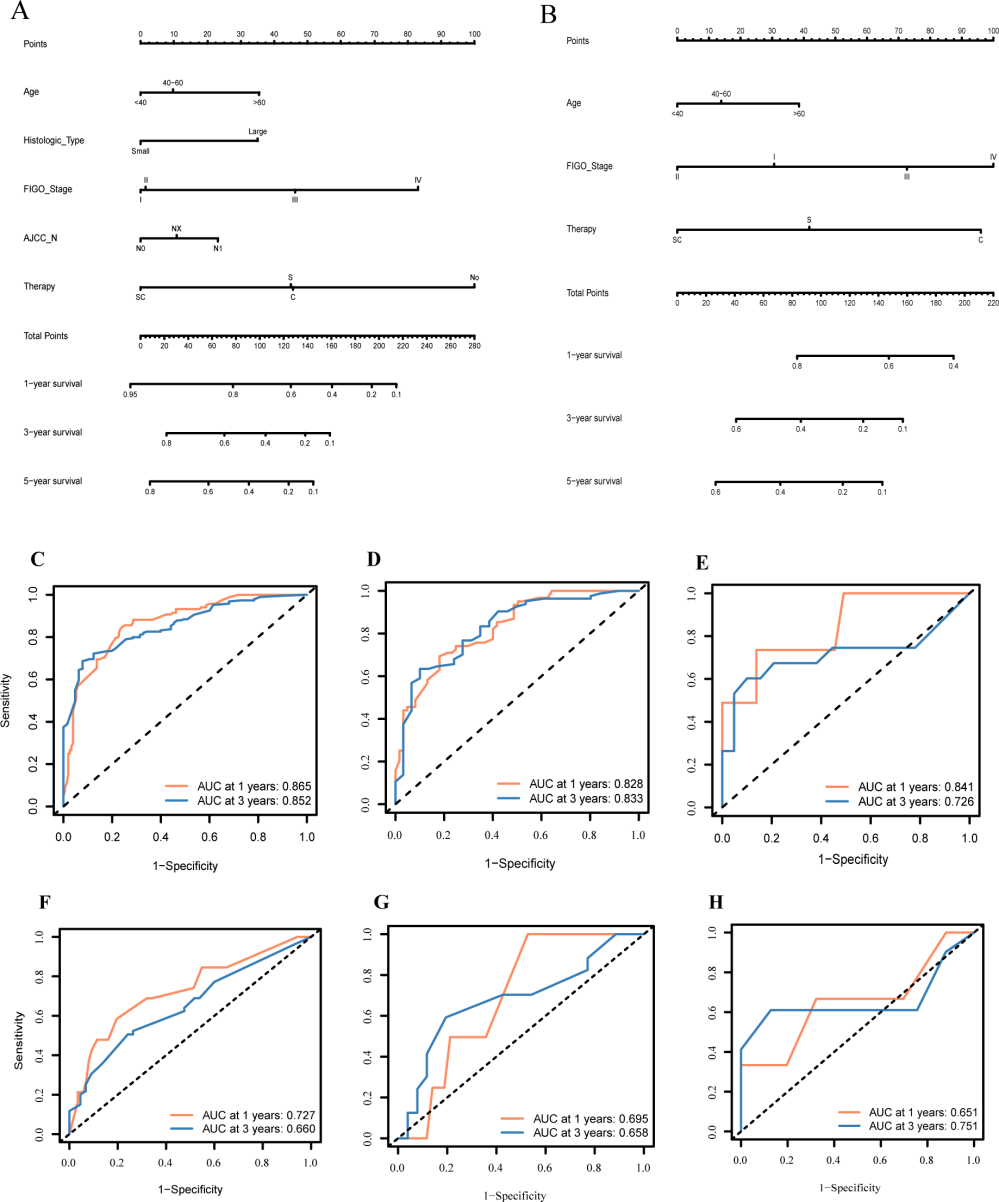
Constructing and validating the two nomograms based on therapeutic differences: **(A)** nomogram of patients without radiotherapy; **(B)** nomogram of patients with radiotherapy; **(C–H)** Receiver operating characteristic curve (ROC) of predictive models for 1- and 3- years in NR-group and R-group individually.

Based on the training group of 199 samples, we conducted Cox regression analysis (**Table 3**). Multi-variable Cox analysis showed that age, histological type, FIGO stage, treatment regimen, and lymph node status were independent risk factors affecting the survival prognosis in NR group (P<0.05). Based on the results, we developed a nomogram model for predicting the 1, 3, and 5-year survival of the NR group and showed the distribution of risk scores for all patients (**Figure 4A**). ROC curves were used to assess the model's discriminative ability, and calibration curves were used to evaluate the consistency between the predicted survival rate and the actual survival rate of the population. Due to the limited number of analyzable samples, including insufficient data for 5-year survival, some groups lack 5-year survival data, we only conducted accuracy assessment for 1 and 3-year survival predictions in this study.

**Table 3:** COX regression analysis of patients without radiotherapy (the training group).

Cox univariate analysis					Cox multivariate analysis				
	HR	HR.95L	HR.95H	P value		HR	HR.95L	HR.95H	P value
Age					Age				
<40	1.000	–	–	–	<40	1.000	–	–	–
40-60	2.218	1.517	3.243	<0.001	40-60	1.168	0.797	1.712	0.426
>60	3.839	2.618	5.629	<0.001	>60	1.820	1.212	2.732	0.004
Primary Site									
Cervix uteri	1.000	–	–	–					
Endocervix/Other	0.944	0.613	1.456	0.796					
Histologic Type					Histologic Type				
Large	1.000	–	–	–	Large	1.000	–	–	–
Small	0.742	0.495	1.113	0.149	Small	0.574	0.369	0.893	0.014
FIGO_Stage					FIGO_Stage				
I	1.000	–	–	–	I	1.000	–	–	–
II	3.141	1.568	6.292	0.001	II	1.046	0.508	2.154	0.904
III	4.116	2.482	6.825	<0.001	III	2.199	1.213	3.985	0.009
IV	8.050	5.001	12.957	<0.001	IV	2.775	1.147	6.714	0.024
AJCC_N					AJCC_N				
N0	1.000	–	–	–	N0	1.000	–	–	–
N1	2.214	1.636	2.996	<0.001	N1	1.500	1.015	2.216	0.042
NX	3.153	2.107	4.719	<0.001	NX	1.196	0.741	1.929	0.463
AJCC_M					AJCC_M				
M0	1.000	–	–	–	M0	1.000	–	–	–
M1	3.311	2.496	4.392	<0.001	M1	1.558	0.743	3.267	0.241
Therapy									
No	1.000	–	–	–	No	1.000	–	–	–
Surgery+Chemotherapy	0.175	0.113	0.272	<0.001	Surgery+Chemotherapy	0.185	0.108	0.319	<0.001
Surgery	0.217	0.126	0.371	<0.001	Surgery	0.395	0.267	0.584	<0.001
Chemotherapy	0.409	0.295	0.566	<0.001	Chemotherapy	0.407	0.214	0.775	0.006

NR, non radiotherapy; R, radiotherapy.

The predictive results for the 1- and 3-year survival in the training group and internal/external validation groups were assessed using ROC curves (**Figures 4C–E**). The model shows prediction accuracy by using the AUC values: In the training group, the AUC values were 0.865 and 0.852, respectively (**Figure 4C**); in the internal validation group, the AUC values were 0.828 and 0.833, respectively (**Figure 4D**). Meanwhile, **Figure 4E** illustrates the analysis of predicting the 1- and 3-year survival accuracy in the external validation group, with AUC values of 0.841 and 0.726, respectively.

Regarding the calibration curve, the X-axis denotes the predicted probability, while the Y-axis represents the actual probability, ranging from 0 to 1. Perfect alignment with the reference line (diagonal line) indicates accurate predictions, while deviation above or below indicates overestimation or underestimation of risk, respectively. Calibration curves for the 1- and 3-year predictive models in the training, internal validation, and external validation groups are depicted (**Supplementary Figure S1**). Notably, all curves fluctuate around the 45-degree dashed line, indicating well-calibrated models.

#### 3.4.2 Construction and validation of nomogram for R-group

Similarly, there were 184 samples in the R group from the SEER database. The sample was randomly divided in a 7:3 ratio by the computer into a training group (115 patients) and an internal validation group (49 patients). Additionally, we considered all 20 patients with radiotherapy from our institution's database as an external validation group (**Supplementary Table S7**). Based on the training group of 115 samples, we conducted Cox regression analysis (**Table 4**). The results of Cox analysis indicated that FIGO stage and treatment regimen were significant factors affecting survival prognosis in the radiotherapy group. The risk of death increased with age, and higher tumor stages were associated with greater mortality risk (P<0.05). Surgery and chemotherapy were found to be protective factors.

**Table 4:** COX regression analysis of patients with radiotherapy (the training group).

COX univariate analysis					COX multivariate analysis				
	HR	HR.95L	HR.95H	P-value		HR	HR.95L	HR.95H	P-value
Age					Age				
<40	1.000	–	–	–	<40	1.000	–	–	–
40-60	1.261	0.762	2.083	0.368	40-60	1.197	0.798	2.044	0.509
>60	1.743	0.919	3.307	0.089	>60	1.649	0.798	3.406	0.177
Primary Site									
Cervix uteri	1.000	–	–	–					
Endo-cervix/Other	1.020	0.601	1.715	0.955					
Histologic Type					Histologic Type				
Large	1.000	–	–	–	Large	1.000	–	–	–
Small	0.889	0.425	1.856	0.754	Small	0.866	0.390	1.922	0.723
FIGO_Stage					FIGO_Stage				
I	1.000	–	–	–	I	1.000	–	–	–
II	0.758	0.265	2.166	0.604	II	0.684	0.228	2.057	0.500
III	1.623	0.960	2.743	0.071	III	3.820	0.797	18.333	0.094
IV	3.064	1.565	5.999	0.001	IV	3.681	1.047	12.950	0.042
AJCC_N					AJCC_N				
N0	1.000	–	–	–	N0	1.000	–	–	–
N1	1.689	1.046	2.729	0.032	N1	0.448	0.103	1.948	0.284
NX	2.553	0.991	6.580	0.052	NX	0.790	0.175	3.580	0.760
AJCC_M									
M0	1.000	–	–	–					
M1	1.927	1.072	3.464	0.028					
Therapy					Therapy				
Surgery+Chemotherapy	1.000	–	–	–		1.000	–	–	–
Surgery	1.728	0.942	3.168	0.077	Surgery	1.718	0.868	3.402	0.124
Chemotherapy 5.470		2.282	13.111	<0.001	Chemotherapy	4.443	1.539	12.828	0.006

Based on the results of the Cox analysis, we constructed a prognostic nomogram for 1-, 3-, and 5-year survival (**Figure 4B**). The analysis included variables such as age, FIGO stage, and treatment regimen. Using R software, we generated risk charts for 1-, 3- and 5-year survival of the NECC-R group patients, as well as scatter plots showing the distribution of risk scores for all patients.

We validated the 1- and 3-year prediction results of the training group, internal validation group, and external validation group by using ROC curves (**Figures 4F–H**). Across all models (based on all clinical and pathological factors), the Cox model's AUCs for the 1- and 3-year OS in the training group was 0.727 and 0.660, respectively (**Figure 4F**). For the internal validation group, the COX model's AUCs for the 1- and 3-year OS was 0.695 and 0.658, respectively (**Figure 4G**). As for the external validation group, the AUC value was 0.651 and 0.751, respectively (**Figure 4H**).

Furthermore, we assessed the calibration of the 1- and 3-year survival prediction models for the three groups using calibration curves (**Supplementary Figure S2**). The curves for predicting the 1- and 3-year survival rates of all three groups fluctuated around the 45-degree line, indicating good calibration performance of the models.

Here, we will demonstrate the application process of the model through two supplementary examples (see in **Supplementary Materials**).

## 4 Discussion

In recent years, as the incidence of NECC continues to rise, research on this disease has also increased. To our knowledge, this is not the first study to examine the clinical characteristics and prognosis of NECC using the SEER database (18–20), That said, however, our study's focus was on investigating the effectiveness of radiotherapy in treating NECC. We aimed to provide more scientifically based clinical guidance for future treatment decisions by quantifying the benefits of radiotherapy and categorizing patients into subgroups based on their radiotherapy status. We believe that this approach is innovative.

In our analysis, we found that the disease tends to affect patients at a younger age and is highly aggressive, with early occurrences of local and distant metastases leading to poor OS. Effective treatments to improve OS rates include surgery and chemotherapy (P<0.05). Cox analysis did not show clear survival benefits from adding radiotherapy. Moreover, the subgroup analysis based on radiotherapy status, after PSM, also did not reveal any significant survival differences between the R and NR groups. However, the radiotherapy group showed a relatively unfavorable survival trend overall (**Figure 2**), similar to findings from previous studies on neuroendocrine carcinoma of the cervix (7, 8, 17, 21, 22). Analyzing the reason, this may be because of the tumor's aggressive nature and early metastasis, which make chemotherapy more beneficial as a systemic treatment (5, 7, 8, 23). Radiation therapy primarily targets local tumor tissues and may not effectively control distant metastases. Additionally, the combined toxic side effects of chemo-radiotherapy could outweigh its benefits for local tumor lesions, leading to shorter survival, as seen in previous studies (6, 8–10).

In our analysis, we found that radiotherapy showed statistically significant survival benefits in NECC patients in the Unix-variable Cox analysis (**Table 2**) and before PSM (**Figure 2**) (P<0.05). This suggests that certain factors in our analysis may maximize the effectiveness of radiotherapy. Radiotherapy can delay the progression and distant metastasis of lesions locally, surpassing the toxic side effects of treatment itself through combined chemo-radiotherapy, thereby prolonging patients' PFS and OS. Recent studies on radiotherapy response in NECC patients have provided insights. Roy et al. (12) studied 25 NECC patients treated with combined chemo-radiotherapy and observed improved OS, with a median survival time of 53.8 months and a 5-year OS rate of 48%. Unfortunately, this study did not further analyze the characteristics of patients who benefited from radiotherapy. Xie et al. (13) analyzed 48 NECC patients' post-surgery and found that the tumor's histological type influenced radiotherapy efficacy. They noted better survival outcomes in neuroendocrine tumors with mixed glandular tissue. Additionally, while early studies mainly focused on adverse effects of radiotherapy in early-stage NECC, recent findings suggest potential benefits in middle and late-stage patients (15, 16).

Our study aimed to identify factors influencing the effectiveness of radiotherapy in patients with HGNECC. The histological type of NECC showed poor response to radiotherapy, especially in SCNEC, which exhibited worse OS outcomes with radiotherapy (P=0.031, **Figure 3**). In early-stage, lymph node-negative, and non-metastatic NECC, patients who did not receive radiotherapy had significantly better OS than those who did. However, in patients with lymph node metastasis (N1), radiotherapy showed clear benefits, significantly improving OS compared to those without radiotherapy (P=0.045). Radiotherapy exhibited excellent local control in patients with N1, reducing the size of the primary lesion to some extent and delaying further invasion of local lymph nodes into distant areas, thereby prolonging PFS and OS. For patients with distant metastasis (M1), while there was a trend of survival benefit with radiotherapy, it didn't reach statistical significance in our study (P = 0.064). However, in a recent research (24):radiotherapy was identified as the only independent prognostic risk factor in the M1 group. There was a statistically significant benefit for OS in the group combined with radiotherapy (with median survival times of 44.6 months and 80.9 months, respectively, p = 0.004). This study suggests that even in the presence of distant metastasis, aggressive local treatment should be considered. These findings underscore the importance of considering individual patient characteristics when determining the role of radiotherapy in HGNECC.

Our study explored factors affecting radiotherapy's effectiveness, confirming and expanding upon previous findings from smaller studies. However, individual patient responses to radiotherapy are influenced by multiple factors, making it challenging to determine treatment solely based on one factor. To address this, we further investigated by grouping patients based on receipt of radiotherapy to create survival prognosis models. To our knowledge, this is the first study to create subgroup nomograms based on treatment differences. Our analyses showed that factors like age, cancer type, disease stage, and treatment method significantly influenced survival in patients not receiving radiotherapy. Similarly, for those receiving radiotherapy, age, disease stage, and treatment method played significant roles. However, given the sample size and data limitations, the accuracy of models in the radiotherapy group was somewhat lower than in the non-radiotherapy group.

### 4.1 Strengths and significance

1. Our study, based on the extensive SEER database, provides more robust and generalizable findings compared to previous small-scale studies on NECC 2. We address the ongoing debate surrounding radiotherapy in the treatment of NECC, offering valuable insights and guidance for clinical decision-making 3.Despite not being the first SEER-based study on NECC, our research stands out by focusing on the efficacy of RT, offering innovative directions for future treatment strategies.

### 4.2 Limitations and future recommendations

1. We know that, for cervical cancer, external irradiation and internal radiation are both used. Although our study discussed the benefits of radiotherapy in patients with HGNECC, there as only "yes, no, or unknown" on radiotherapy information based on the SEER database. Our hospital database only includes 20 cases of radiotherapy patients. Due to the small amount of analyzable data and the limitations imposed by recall bias from patients, the detailed radiotherapy regimens in this study cannot be specifically shown. Therefore, we will further investigate the efficacy of radiotherapy in patients and explore detailed radiotherapy regimens by making more accurate prospective studies. 2. Missing data on crucial factors like postoperative pathology and specific treatment regimens limit the accuracy of our analyses, suggesting the need for more comprehensive data collection methods. 3. We primarily focuse on survival outcomes, overlooking aspects like recurrence and treatment-related adverse events. 4. Future research should delve deeper into these areas; for a more comprehensive assessment of RT efficacy, additional comparison indicators and validation from other hospital databases are recommended.

### 4.3 Conclusion

In HGNECC, radiotherapy can result in survival benefits for patients with positive lymph nodes. Patients with distant metastases should also be considered for appropriate. We have established two specific nomograms to predict treatment outcomes, helping doctors plan personalized treatments.

## Data Availability

The datasets presented in this study can be found in online repositories. The names of the repository/repositories and accession number(s) can be found in the article/**Supplementary Material**.
