# Supplementary material for: Radiotherapy can improve overall survival in patients with lymph-node positive, high-grade neuroendocrine cervical cancer: construction of two prognostic nomograms to predict treatment outcome
Source: Front Oncol. 2024 Sep 13;14:1450382. doi: 10.3389/fonc.2024.1450382 (PMC11427233; doi:10.3389/fonc.2024.1450382)
Supplement: Supplementary file 1 [file DataSheet1.docx]

Supplementary Material

# Supplementary Figures and Tables

## 1.1 Supplementary Figures


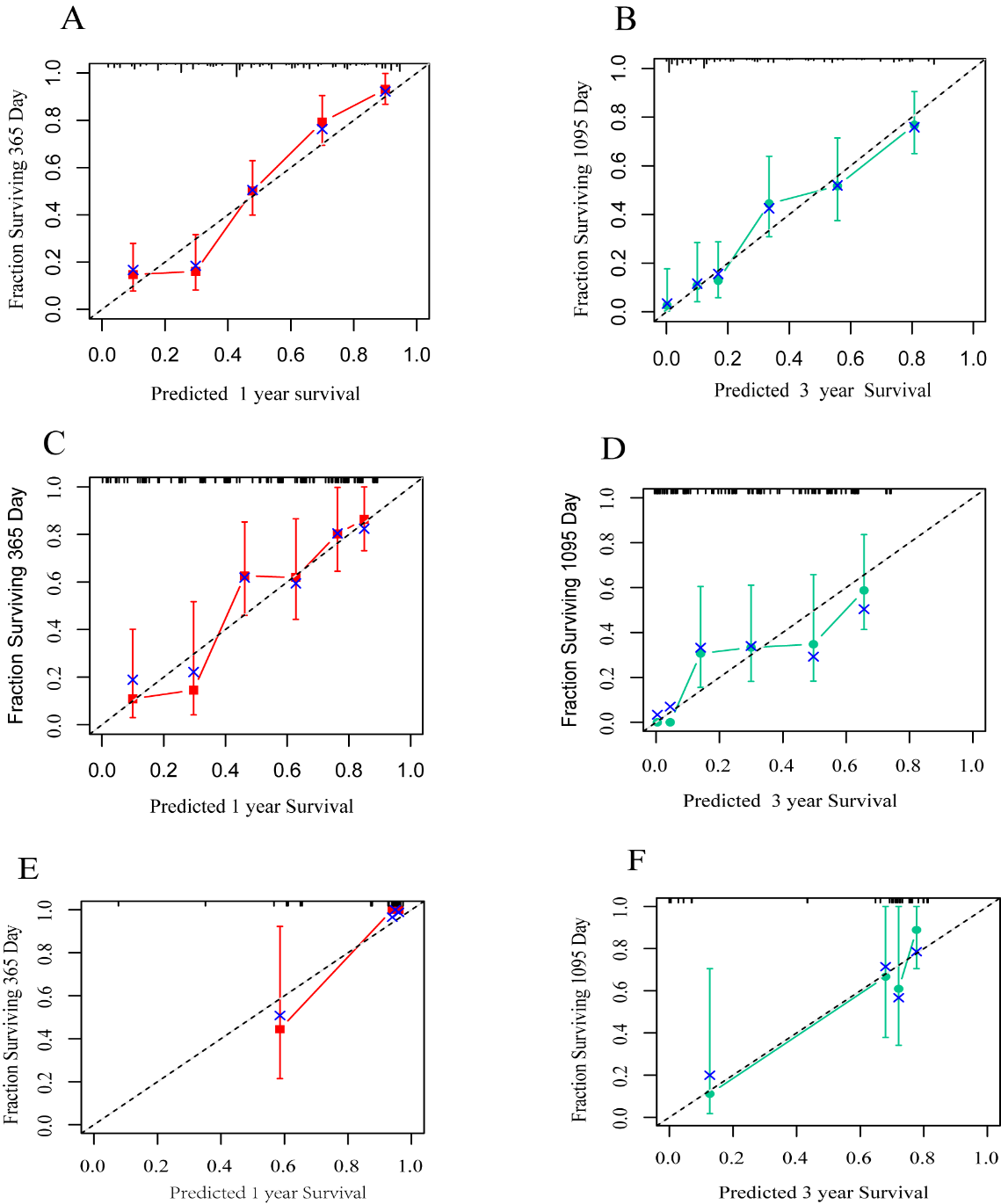


Figure S1. Accuracy verification of the prediction ability of the survival model by the Calibration Curve: A/B Training group; C/D Internal validation group; E/F External validation group

##
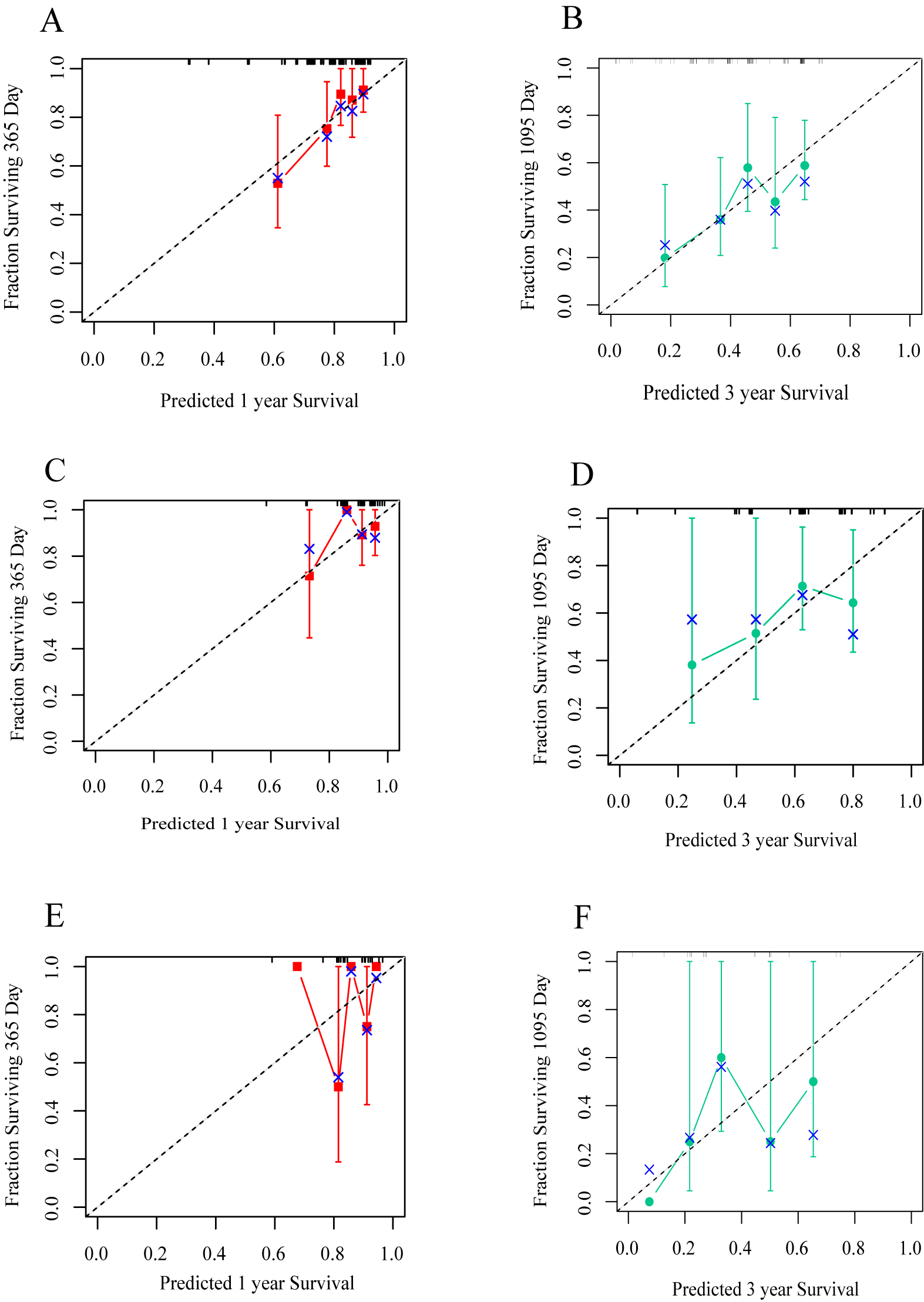


Figure S2. Accuracy verification of the prediction ability of the survival model by the Calibration Curve: A/B Training group; C/D Internal validation group; E/F External validation group

## 1.2 Supplementary Tables

**Table S1. Baseline data of all enrolled patients from SEER database before and after PSM**

| Before PSM | | | | After PSM | | | |
| --- | --- | --- | --- | --- | --- | --- | --- |
|  | NR  (N=427) | R  (N=164) | P-value |  | NR  (N=100) | R  (N=100) | P-value |
| Age |  |  |  | Age |  |  |  |
| <40 | 112 (26.2%) | 82 (50.0%) | <0.001 | <40 | 49 (49.0%) | 58 (58.0%) | 0.153 |
| >60 | 138 (32.3%) | 19 (11.6%) |  | >60 | 18 (18.0%) | 9 (9.0%) |  |
| 40-60 | 177 (41.5%) | 63 (38.4%) |  | 40-60 | 33 (33.0%) | 33 (33.0%) |  |
| Primary Site | |  |  | Primary Site | |  |  |
| Cervix uteri | 380 (89.0%) | 123 (75.0%) | <0.001 | Cervix uteri | 86 (86.0%) | 76 (76.0%) | 0.105 |
| Endo-Cervix/ other | 47 (11.0%) | 41 (25.0%) |  | Endo-Cervix/ other | 14 (14.0%) | 24 (24.0%) |  |
| Histologic Type | |  |  | Histologic Type | |  |  |
| Large | 47 (11.0%) | 17 (10.4%) | 0.939 | Large | 20 (20.0%) | 16 (16.0%) | 0.581 |
| Small | 380 (89.0%) | 147 (89.6%) |  | Small | 80 (80.0%) | 84 (84.0%) |  |
| FIGO_Stage | |  |  | FIGO_Stage | |  |  |
| I | 90 (21.1%) | 66 (40.2%) | <0.001 | I | 46 (46.0%) | 46 (46.0%) | 1 |
| II | 33 (7.7%) | 12 (7.3%) |  | II | 1 (1.0%) | 1 (1.0%) |  |
| III | 98 (23.0%) | 65 (39.6%) |  | III | 32 (32.0%) | 32 (32.0%) |  |
| IV | 206 (48.2%) | 21 (12.8%) |  | IV | 21 (21.0%) | 21 (21.0%) |  |
| AJCC_N |  |  |  | AJCC_N |  |  |  |
| N0 | 186 (43.6%) | 83 (50.6%) | 0.006 | N0 | 55 (55.0%) | 52 (52.0%) | 0.910 |
| N1 | 185 (43.3%) | 74 (45.1%) |  | N1 | 40 (40.0%) | 43 (43.0%) |  |
| NX | 56 (13.1%) | 7 (4.3%) |  | NX | 5 (5.0%) | 5 (5.0%) |  |
| AJCC_M |  |  |  | AJCC_M |  |  |  |
| M0 | 232 (54.3%) | 141 (86.0%) | <0.001 | M0 | 80 (80.0%) | 79 (79.0%) | 1 |
| M1 | 195 (45.7%) | 23 (14.0%) |  | M1 | 20 (20.0%) | 21 (21.0%) |  |
| Surgery |  |  |  | Surgery |  |  |  |
| No | 300 (70.3%) | 9 (5.5%) | <0.001 | No | 9 (9.0%) | 9 (9.0%) | 1 |
| Yes | 127 (29.7%) | 155 (94.5%) |  | Yes | 91 (91.0%) | 91 (91.0%) |  |
| Chemotherapy | |  |  | Chemotherapy | |  |  |
| No/Unknown | 122 (28.6%) | 18 (11.0%) | <0.001 | No/Unknown | 16 (16.0%) | 16 (16.0%) | 1 |
| Yes | 305 (71.4%) | 146 (89.0%) |  | Yes | 84 (84.0%) | 84 (84.0%) |  |

**Abbreviation:** PSM, Propensity score matching; R, radiotherapy; NR, non-radiotherapy

**Table S2.1 Baseline data of enrolled patients from SEER database with FIGO I-II before and after PSM**

| **Before PSM** | | | | **After PSM** | | | |
| --- | --- | --- | --- | --- | --- | --- | --- |
|  | **NR** | **R** | **P-value** |  | **NR** | **R** | **P-value** |
|  | **(N=123)** | **(N=78)** |  |  | **(N=47)** | **(N=47)** |  |
| **Age** |  |  |  | **Age** |  |  |  |
| <40 | 48 (39.0%) | 38 (48.7%) | 0.298 | <40 | 25 (53.2%) | 28 (59.6%) | 0.778 |
| >60 | 28 (22.8%) | 12 (15.4%) |  | >60 | 7 (14.9%) | 7 (14.9%) |  |
| 40-60 | 47 (38.2%) | 28 (35.9%) |  | 40-60 | 15 (31.9%) | 12 (25.5%) |  |
| **Primary Site** | |  |  | **Primary Site** | |  |  |
| Cervix uteri | 105 (85.4%) | 56 (71.8%) | 0.0302 | Cervix uteri | 40 (85.1%) | 34 (72.3%) | 0.208 |
| Endo-cervix/Other | 18 (14.6%) | 22 (28.2%) |  | Endo-cervix/Other | 7 (14.9%) | 13 (27.7%) |  |
| **Histologic Type** | |  |  | **Histologic Type** | |  |  |
| Large | 11 (8.9%) | 10 (12.8%) | 0.523 | Large | 9 (19.1%) | 10 (21.3%) | 1 |
| Small | 112 (91.1%) | 68 (87.2%) |  | Small | 38 (80.9%) | 37 (78.7%) |  |
| **AJCC_N** |  |  |  | **AJCC_N** |  |  |  |
| N0 | 121 (98.4%) | 77 (98.7%) | 1 | N0 | 47 (100%) | 46 (97.9%) | 1 |
| NX | 2 (1.6%) | 1 (1.3%) |  | NX | 0 (0%) | 1 (2.1%) |  |
| **AJCC_M** |  |  |  | **AJCC_M** |  |  |  |
| M0 | 123 (100%) | 78 (100%) | 0.0015 | M0 | 47 (100%) | 47 (100%) | 1 |
| **Surgery** |  |  |  | **Surgery** |  |  |  |
| No | 62 (50.4%) | 0 (0%) | <0.001 | No | 0 (0%) | 0 (0%) |  |
| Yes | 61 (49.6%) | 78 (100%) |  | Yes | 47 (100%) | 47 (100%) | 1 |
| **Chemotherapy** | |  |  | **Chemotherapy** | |  |  |
| No/Unknown | 36 (29.3%) | 10 (12.8%) | 0.0113 | No/Unknown | 8 (17.0%) | 8 (17.0%) | 1 |
| Yes | 87 (70.7%) | 68 (87.2%) |  | Yes | 39 (83.0%) | 39 (83.0%) |  |

**Table S2.2 Baseline data of enrolled patients from SEER database with FIGO III-IV before and after PSM**

| **Before PSM** | | | | **After PSM** | | | | |
| --- | --- | --- | --- | --- | --- | --- | --- | --- |
|  | **NR** | **R** | **P-value** | |  | **NR** | **R** | **P-value** |
|  | **(N=304)** | **(N=86)** |  |  |  | **(N=54)** | **(N=54)** |  |
| **Age** |  |  |  | | **Age** |  |  |  |
| <40 | 64 (21.1%) | 44 (51.2%) | <0.001 | | <40 | 28 (51.9%) | 29 (53.7%) | 0.978 |
| >60 | 110 (36.2%) | 7 (8.1%) |  | | >60 | 7 (13.0%) | 7 (13.0%) |  |
| 40-60 | 130 (42.8%) | 35 (40.7%) |  | | 40-60 | 19 (35.2%) | 18 (33.3%) |  |
| **Primary Site** | |  |  | | **Primary Site** | |  |  |
| Cervix uteri | 275 (90.5%) | 67 (77.9%) | 0.003 | | Cervix uteri | 44 (81.5%) | 43 (79.6%) | 1 |
| Endo-cervix/Other | 29 (9.5%) | 19 (22.1%) |  | | Endo-cervix/Other | 10 (18.5%) | 11 (20.4%) |  |
| **Histologic Type** | |  |  | | **Histologic Type** | |  |  |
| Large | 36 (11.8%) | 7 (8.1%) | 0.44 | | Large | 12 (22.2%) | 6 (11.1%) | 0.197 |
| Small | 268 (88.2%) | 79 (91.9%) |  | | Small | 42 (77.8%) | 48 (88.9%) |  |
| **AJCC_N** |  |  |  | | **AJCC_N** |  |  |  |
| N0 | 65 (21.4%) | 6 (7.0%) | <0.001 | | N0 | 12 (22.2%) | 5 (9.3%) | 0.178 |
| N1 | 185 (60.9%) | 74 (86.0%) |  | | N1 | 39 (72.2%) | 45 (83.3%) |  |
| NX | 54 (17.8%) | 6 (7.0%) |  | | NX | 3 (5.6%) | 4 (7.4%) |  |
| **AJCC_M** |  |  |  | | **AJCC_M** |  |  |  |
| M0 | 109 (35.9%) | 63 (73.3%) | <0.001 | | M0 | 28 (51.9%) | 37 (68.5%) | 0.116 |
| M1 | 195 (64.1%) | 23 (26.7%) |  | | M1 | 26 (48.1%) | 17 (31.5%) |  |
| **Surgery** |  |  |  | | **Surgery** |  |  |  |
| No | 238 (78.3%) | 9 (10.5%) | <0.001 | | No | 9 (16.7%) | 9 (16.7%) | 1 |
| Yes | 66 (21.7%) | 77 (89.5%) |  | | Yes | 45 (83.3%) | 45 (83.3%) |  |
| **Chemotherapy** | |  |  | | **Chemotherapy** | |  |  |
| No/Unknown | 86 (28.3%) | 8 (9.3%) | <0.001 | | No/Unknown | 6 (11.1%) | 7 (13.0%) | 1 |
| Yes | 218 (71.7%) | 78 (90.7%) |  | | Yes | 48 (88.9%) | 47 (87.0%) |  |

**Table S3.1 Baseline data of enrolled patients from SEER database with negative lymph node (N0) before and after PSM**

| **Before PSM** | | | | **After PSM** | | | |
| --- | --- | --- | --- | --- | --- | --- | --- |
|  | **NR** | **R** | **P-value** |  | **NR** | **R** | **P-value** |
|  | **(N=186)** | **(N=83)** |  |  | **(N=53)** | **(N=53)** |  |
| **Age** |  |  |  | **Age** |  |  |  |
| <40 | 58 (31.2%) | 40 (48.2%) | 0.006 | <40 | 28 (52.8%) | 31 (58.5%) | 0.842 |
| >60 | 56 (30.1%) | 12 (14.5%) |  | >60 | 8 (15.1%) | 7 (13.2%) |  |
| 40-60 | 72 (38.7%) | 31 (37.3%) |  | 40-60 | 17 (32.1%) | 15 (28.3%) |  |
| **Primary Site** | |  |  | **Primary Site** | |  |  |
| Cervix uteri | 163 (87.6%) | 61 (73.5%) | 0.007 | Cervix uteri | 45 (84.9%) | 40 (75.5%) | 0.33 |
| Endo-cervix/Other | 23 (12.4%) | 22 (26.5%) |  | Endo-cervix/Other | 8 (15.1%) | 13 (24.5%) |  |
| **Histologic Type** | |  |  | **Histologic Type** | |  |  |
| Large | 19 (10.2%) | 9 (10.8%) | 1 | Large | 10 (18.9%) | 9 (17.0%) | 1 |
| Small | 167 (89.8%) | 74 (89.2%) |  | Small | 43 (81.1%) | 44 (83.0%) |  |
| **FIGO_Stage** | |  |  | **FIGO_Stage** | |  |  |
| I | 89 (47.8%) | 65 (78.3%) | <0.001 | I | 46 (86.8%) | 46 (86.8%) | 1 |
| II | 32 (17.2%) | 12 (14.5%) |  | II | 1 (1.9%) | 1 (1.9%) |  |
| III | 22 (11.8%) | 1 (1.2%) |  | III | 1 (1.9%) | 1 (1.9%) |  |
| IV | 43 (23.1%) | 5 (6.0%) |  | IV | 5 (9.4%) | 5 (9.4%) |  |
| **AJCC_M** |  |  |  | **AJCC_M** |  |  |  |
| M0 | 146 (78.5%) | 78 (94.0%) | 0.003 | M0 | 48 (90.6%) | 48 (90.6%) | 1 |
| M1 | 40 (21.5%) | 5 (6.0%) |  | M1 | 5 (9.4%) | 5 (9.4%) |  |
| **Surgery** |  |  |  | **Surgery** |  |  |  |
| No | 113 (60.8%) | 2 (2.4%) | <0.001 | No | 2 (3.8%) | 2 (3.8%) | 1 |
| Yes | 73 (39.2%) | 81 (97.6%) |  | Yes | 51 (96.2%) | 51 (96.2%) |  |
| **Chemotherapy** | |  |  | **Chemotherapy** | |  |  |
| No/Unknown | 59 (31.7%) | 10 (12.0%) | 0.001 | No/Unknown | 8 (15.1%) | 8 (15.1%) | 1 |
| Yes | 127 (68.3%) | 73 (88.0%) |  | Yes | 45 (84.9%) | 45 (84.9%) |  |

**Table S3.2 Baseline data of enrolled patients from SEER database with positive lymph node (N1) before and after PSM**

| **Before PSM** | | | | **After PSM** | | | |
| --- | --- | --- | --- | --- | --- | --- | --- |
|  | **NR** | **R** | **P-value** |  | **NR** | **R** | **P-value** |
|  | **(N=185)** | **(N=74)** |  |  | **(N=43)** | **(N=43)** |  |
| **Age** |  |  |  | **Age** |  |  |  |
| <40 | 47 (25.4%) | 39 (52.7%) | <0.001 | <40 | 24 (55.8%) | 21 (48.8%) | 0.549 |
| >60 | 60 (32.4%) | 6 (8.1%) |  | >60 | 3 (7.0%) | 6 (14.0%) |  |
| 40-60 | 78 (42.2%) | 29 (39.2%) |  | 40-60 | 16 (37.2%) | 16 (37.2%) |  |
| **Primary Site** | |  |  | **Primary Site** | |  |  |
| Cervix uteri | 164 (88.6%) | 55 (74.3%) | 0.00711 | Cervix uteri | 35 (81.4%) | 34 (79.1%) | 1 |
| Endo-cervix/Other | 21 (11.4%) | 19 (25.7%) |  | Endo-cervix/Other | 8 (18.6%) | 9 (20.9%) |  |
| **Histologic Type** | |  |  | **Histologic Type** | |  |  |
| Large | 27 (14.6%) | 7 (9.5%) | 0.367 | Large | 10 (23.3%) | 6 (14.0%) | 0.406 |
| Small | 158 (85.4%) | 67 (90.5%) |  | Small | 33 (76.7%) | 37 (86.0%) |  |
| **FIGO_Stage** | |  |  | **FIGO_Stage** | |  |  |
| III | 73 (39.5%) | 62 (83.8%) | <0.001 | III | 24 (55.8%) | 32 (74.4%) | 0.113 |
| IV | 112 (60.5%) | 12 (16.2%) |  | IV | 19 (44.2%) | 11 (25.6%) |  |
| **AJCC_M** |  |  |  | **AJCC_M** |  |  |  |
| M0 | 79 (42.7%) | 60 (81.1%) | <0.001 | M0 | 24 (55.8%) | 33 (76.7%) | 0.068 |
| M1 | 106 (57.3%) | 14 (18.9%) |  | M1 | 19 (44.2%) | 10 (23.3%) |  |
| **Surgery** |  |  |  | **Surgery** |  |  |  |
| No | 138 (74.6%) | 7 (9.5%) | <0.001 | No | 7 (16.3%) | 7 (16.3%) | 1 |
| Yes | 47 (25.4%) | 67 (90.5%) |  | Yes | 36 (83.7%) | 36 (83.7%) |  |
| **Chemotherapy** | |  |  | **Chemotherapy** | |  |  |
| No/Unknown | 40 (21.6%) | 7 (9.5%) | 0.0344 | No/Unknown | 8 (18.6%) | 5 (11.6%) | 0.547 |
| Yes | 145 (78.4%) | 67 (90.5%) |  | Yes | 35 (81.4%) | 38 (88.4%) |  |

**Table S4.1 Baseline data of enrolled patients from SEER database without metastasis (M0) before and after PSM**

| **Before PSM** | | | | **After PSM** | | | |
| --- | --- | --- | --- | --- | --- | --- | --- |
|  | **NR** | **R** | **P-value** |  | **NR** | **R** | **P-value** |
|  | **(N=232)** | **(N=141)** |  |  | **(N=76)** | **(N=76)** |  |
| **Age** |  |  |  | **Age** |  |  |  |
| <40 | 86 (37.1%) | 68 (48.2%) | <0.001 | <40 | 43 (56.6%) | 41 (53.9%) | 0.943 |
| >60 | 67 (28.9%) | 17 (12.1%) |  | >60 | 10 (13.2%) | 11 (14.5%) |  |
| 40-60 | 79 (34.1%) | 56 (39.7%) |  | 40-60 | 23 (30.3%) | 24 (31.6%) |  |
| **Primary Site** | |  |  | **Primary Site** | |  |  |
| Cervix uteri | 204 (87.9%) | 103 (73.0%) | <0.001 | Cervix uteri | 60 (78.9%) | 58 (76.3%) | 0.846 |
| Endo-Cervix/Other | 28 (12.1%) | 38 (27.0%) |  | Endo-Cervix/Other | 16 (21.1%) | 18 (23.7%) |  |
| **Histologic Type** | |  |  | **Histologic Type** | |  |  |
| Large | 17 (7.3%) | 13 (9.2%) | 0.649 | Large | 14 (18.4%) | 13 (17.1%) | 1 |
| Small | 215 (92.7%) | 128 (90.8%) |  | Small | 62 (81.6%) | 63 (82.9%) |  |
| **FIGO_Stage** | |  |  | **FIGO_Stage** | |  |  |
| I | 90 (38.8%) | 66 (46.8%) | 0.0317 | I | 46 (60.5%) | 45 (59.2%) | 0.598 |
| II | 33 (14.2%) | 12 (8.5%) |  | II | 1 (1.3%) | 3 (3.9%) |  |
| III | 97 (41.8%) | 62 (44.0%) |  | III | 29 (38.2%) | 28 (36.8%) |  |
| IV | 12 (5.2%) | 1 (0.7%) |  | IV | 0 (0%) | 0 (0%) |  |
| **AJCC_N** |  |  |  | **AJCC_N** |  |  |  |
| N0 | 146 (62.9%) | 78 (55.3%) | 0.246 | N0 | 49 (64.5%) | 48 (63.2%) | 0.214 |
| N1 | 79 (34.1%) | 60 (42.6%) |  | N1 | 27 (35.5%) | 25 (32.9%) |  |
| NX | 7 (3.0%) | 3 (2.1%) |  | NX | 0 (0%) | 3 (3.9%) |  |
| **Surgery** |  |  |  | **Surgery** |  |  |  |
| No | 140 (60.3%) | 4 (2.8%) | <0.001 | No | 4 (5.3%) | 4 (5.3%) | 1 |
| Yes | 92 (39.7%) | 137 (97.2%) |  | Yes | 72 (94.7%) | 72 (94.7%) |  |
| **Chemotherapy** | |  |  | **Chemotherapy** | |  |  |
| No/Unknown | 68 (29.3%) | 16 (11.3%) | <0.001 | No/Unknown | 12 (15.8%) | 15 (19.7%) | 0.671 |
| Yes | 164 (70.7%) | 125 (88.7%) |  | Yes | 64 (84.2%) | 61 (80.3%) |  |

**Table S4.2 Baseline data of enrolled patients from SEER database with metastasis (M1) before and after PSM**

| **Before PSM** | | | | **After PSM** | | | |
| --- | --- | --- | --- | --- | --- | --- | --- |
|  | **NR** | **R** | **P-value** |  | **NR** | **R** | **P-value** |
|  | **(N=195)** | **(N=23)** |  |  | **(N=19)** | **(N=19)** |  |
| **Age** |  |  |  | **Age** |  |  |  |
| <40 | 26 (13.3%) | 14 (60.9%) | <0.001 | <40 | 10 (52.6%) | 10 (52.6%) | 1 |
| >60 | 71 (36.4%) | 2 (8.7%) |  | >60 | 2 (10.5%) | 2 (10.5%) |  |
| 40-60 | 98 (50.3%) | 7 (30.4%) |  | 40-60 | 7 (36.8%) | 7 (36.8%) |  |
| **Primary Site** | |  |  | **Primary Site** | |  |  |
| Cervix uteri | 176 (90.3%) | 20 (87.0%) | 0.896 | Cervix uteri | 15 (78.9%) | 17 (89.5%) | 0.656 |
| Endo-Cervix/Other | 19 (9.7%) | 3 (13.0%) |  | Endo-Cervix/Other | 4 (21.1%) | 2 (10.5%) |  |
| **Histologic Type** | |  |  | **Histologic Type** | |  |  |
| Large | 30 (15.4%) | 4 (17.4%) | 1 | Large | 6 (31.6%) | 4 (21.1%) | 0.713 |
| Small | 165 (84.6%) | 19 (82.6%) |  | Small | 13 (68.4%) | 15 (78.9%) |  |
| **AJCC_N** |  |  |  | **AJCC_N** |  |  |  |
| N0 | 40 (20.5%) | 5 (21.7%) | 0.712 | N0 | 4 (21.1%) | 4 (21.1%) | 0.659 |
| N1 | 106 (54.4%) | 14 (60.9%) |  | N1 | 11 (57.9%) | 13 (68.4%) |  |
| NX | 49 (25.1%) | 4 (17.4%) |  | NX | 4 (21.1%) | 2 (10.5%) |  |
| **Surgery** |  |  |  | **Surgery** |  |  |  |
| No | 160 (82.1%) | 5 (21.7%) | <0.001 | No | 5 (26.3%) | 5 (26.3%) | 1 |
| Yes | 35 (17.9%) | 18 (78.3%) |  | Yes | 14 (73.7%) | 14 (73.7%) |  |
| **Chemotherapy** | |  |  | **Chemotherapy** | |  |  |
| No/Unknown | 54 (27.7%) | 2 (8.7%) | 0.0855 | No/Unknown | 3 (15.8%) | 1 (5.3%) | 0.597 |
| Yes | 141 (72.3%) | 21 (91.3%) |  | Yes | 16 (84.2%) | 18 (94.7%) |  |
| **FIGO_Stage** |  |  |  | **FIGO_Stage** |  |  |  |
| IV | 195 (100%) | 23 (100%) |  | IV | 19 (100%) | 19 (100%) |  |

**Table S5.1 Baseline data of SNECC patients from SEER database before and after PSM**

| **Before PSM** | | | | **After PSM** | | | |
| --- | --- | --- | --- | --- | --- | --- | --- |
|  | **NR** | **R** | **P-value** |  | **NR** | **R** | **P-value** |
|  | **(N=380)** | **(N=147)** |  |  | **(N=77)** | **(N=77)** |  |
| **Age** |  |  |  | **Age** |  |  |  |
| <40 | 99 (26.1%) | 74 (50.3%) | <0.001 | <40 | 41 (53.2%) | 41 (53.2%) | 1 |
| >=40 | 281 (73.9%) | 73 (49.7%) |  | >=40 | 36 (46.8%) | 36 (46.8%) |  |
| **Primary Site** | |  |  | **Primary Site** | |  |  |
| Cervix uteri | 340 (89.5%) | 108 (73.5%) | <0.001 | Cervix uteri | 66 (85.7%) | 58 (75.3%) | 0.154 |
| Endo-cervix/Other | 40 (10.5%) | 39 (26.5%) |  | Endocervix/Other | 11 (14.3%) | 19 (24.7%) |  |
| **FIGO_Stage** | |  |  | **FIGO_Stage** | |  |  |
| I | 81 (21.3%) | 57 (38.8%) | <0.001 | I | 38 (49.4%) | 36 (46.8%) | 0.561 |
| II | 31 (8.2%) | 11 (7.5%) |  | II | 0 (0%) | 2 (2.6%) |  |
| III | 91 (23.9%) | 61 (41.5%) |  | III | 25 (32.5%) | 25 (32.5%) |  |
| IV | 177 (46.6%) | 18 (12.2%) |  | IV | 14 (18.2%) | 14 (18.2%) |  |
| **AJCC_N** |  |  |  | **AJCC_N** |  |  |  |
| N0 | 167 (43.9%) | 74 (50.3%) | 0.00364 | N0 | 45 (58.4%) | 42 (54.5%) | 0.733 |
| N1 | 158 (41.6%) | 67 (45.6%) |  | N1 | 29 (37.7%) | 30 (39.0%) |  |
| NX | 55 (14.5%) | 6 (4.1%) |  | NX | 3 (3.9%) | 5 (6.5%) |  |
| **AJCC_M** |  |  |  | **AJCC_M** |  |  |  |
| M0 | 215 (56.6%) | 128 (87.1%) | <0.001 | M0 | 64 (83.1%) | 64 (83.1%) | 1 |
| M1 | 165 (43.4%) | 19 (12.9%) |  | M1 | 13 (16.9%) | 13 (16.9%) |  |
| **Surgery** |  |  |  | **Surgery** |  |  |  |
| No | 275 (72.4%) | 8 (5.4%) | <0.001 | No | 8 (10.4%) | 8 (10.4%) | 1 |
| Yes | 105 (27.6%) | 139 (94.6%) |  | Yes | 69 (89.6%) | 69 (89.6%) |  |
| **Chemotherapy** | |  |  | **Chemotherapy** | |  |  |
| No/Unknown | 111 (29.2%) | 16 (10.9%) | <0.001 | No/Unknown | 11 (14.3%) | 13 (16.9%) | 0.824 |
| Yes | 269 (70.8%) | 131 (89.1%) |  | Yes | 66 (85.7%) | 64 (83.1%) |  |

**Table S5.2 Baseline data of LNECC patients from SEER database before and after PSM**

| **Before PSM** | | | | **After PSM** | | | |
| --- | --- | --- | --- | --- | --- | --- | --- |
|  | **NR** | **R** | **P-value** |  | **NR** | **R** | **P-value** |
|  | **(N=47)** | **(N=17)** |  |  | **(N=17)** | **(N=17)** |  |
| **Age** |  |  |  | **Age** |  |  |  |
| <40 | 13 (27.7%) | 8 (47.1%) | 0.247 | <40 | 9 (52.9%) | 8 (47.1%) | 1 |
| >=40 | 34 (72.3%) | 9 (52.9%) |  | >=40 | 8 (47.1%) | 9 (52.9%) |  |
| **Primary Site** | |  |  | **Primary Site** | |  |  |
| Cervix uteri | 40 (85.1%) | 15 (88.2%) | 1 | Cervix uteri | 13 (76.5%) | 15 (88.2%) | 0.653 |
| Endo-cervix/Other | 7 (14.9%) | 2 (11.8%) |  | Endo-cervix/Other | 4 (23.5%) | 2 (11.8%) |  |
| **FIGO_Stage** | |  |  | **FIGO_Stage** | |  |  |
| I | 9 (19.1%) | 9 (52.9%) | 0.0146 | I | 5 (29.4%) | 9 (52.9%) | 0.433 |
| II | 2 (4.3%) | 1 (5.9%) |  | II | 1 (5.9%) | 1 (5.9%) |  |
| III | 7 (14.9%) | 4 (23.5%) |  | III | 4 (23.5%) | 4 (23.5%) |  |
| IV | 29 (61.7%) | 3 (17.6%) |  | IV | 7 (41.2%) | 3 (17.6%) |  |
| **AJCC_N** |  |  |  | **AJCC_N** |  |  |  |
| N0 | 19 (40.4%) | 9 (52.9%) | 0.442 | N0 | 8 (47.1%) | 9 (52.9%) | 0.52 |
| N1 | 27 (57.4%) | 7 (41.2%) |  | N1 | 9 (52.9%) | 7 (41.2%) |  |
| NX | 1 (2.1%) | 1 (5.9%) |  | NX | 0 (0%) | 1 (5.9%) |  |
| **AJCC_M** |  |  |  | **AJCC_M** |  |  |  |
| M0 | 17 (36.2%) | 13 (76.5%) | 0.0102 | M0 | 10 (58.8%) | 13(76.5%) | 0.463 |
| M1 | 30 (63.8%) | 4 (23.5%) |  | M1 | 7 (41.2%) | 4 (23.5%) |  |
| **Surgery** |  |  |  | **Surgery** |  |  |  |
| No | 25 (53.2%) | 1 (5.9%) | 0.00184 | No | 1 (5.9%) | 1 (5.9%) | 1 |
| Yes | 22 (46.8%) | 16 (94.1%) |  | Yes | 16 (94.1%) | 16(94.1%) |  |
| **Chemotherapy** | |  |  | **Chemotherapy** | |  |  |
| No/Unknown | 11 (23.4%) | 2 (11.8%) | 0.503 | No/Unknown | 2 (11.8%) | 2 (11.8%) | 1 |
| Yes | 36 (76.6%) | 15 (88.2%) |  | Yes | 15 (88.2%) | 15(88.2%) |  |

**Abbreviation:** PSM, Propensity score matching; LNECC, large-cell neuroendocrine cervical cancer; SNECC, small-cell neuroendocrine cervical cancer.

**Table S6.Baseline characteristics of the training and internal/external-validation datasets in NR group**

|  | **Training Group** | **Internal-validation Group** | **External-validation Group** |
| --- | --- | --- | --- |
|  | **(N=299)** | **(N=128)** | **(N=36)** |
| **Survival time** |  |  |  |
| Mean (SD) | 44.0 (75.7) | 43.0 (77.6) | 38.4 (25.1) |
| Median [Min, Max] | 12.0 [0, 335] | 13.5 [0, 377] | 35.2 [11.2, 95.0] |
| **Age** |  |  |  |
| <40 | 81 (27.1%) | 31 (24.2%) | 7 (19.4%) |
| 40-60 | 116 (38.8%) | 61 (47.7%) | 26 (72.2%) |
| >60 | 102 (34.1%) | 36 (28.1%) | 3 (8.3%) |
| **Primary Site** |  |  |  |
| Cervix uteri | 266 (89.0%) | 114 (89.1%) | 25 (69.4%) |
| Endo-cervix/Other | 33 (11.0%) | 14 (10.9%) | 11 (30.6%) |
| **Histologic Type** |  |  |  |
| Large | 34 (11.4%) | 13 (10.2%) | 18 (50.0%) |
| Small | 265 (88.6%) | 115 (89.8%) | 18 (50.0%) |
| **FIGO_Stage** |  |  |  |
| I | 65 (21.7%) | 25 (19.5%) | 19 (52.8%) |
| II | 19 (6.4%) | 14 (10.9%) | 10 (27.8%) |
| III | 74 (24.7%) | 24 (18.8%) | 6 (16.7%) |
| IV | 141 (47.2%) | 65 (50.8%) | 1 (2.8%) |
| **N** |  |  |  |
| N0 | 130 (43.5%) | 56 (43.8%) | 29 (80.6%) |
| N1 | 130 (43.5%) | 55 (43.0%) | 7 (19.4%) |
| NX | 39 (13.0%) | 17 (13.3%) | 0 (0%) |
| **M** |  |  |  |
| M0 | 168 (56.2%) | 64 (50.0%) | 36 (100%) |
| M1 | 131 (43.8%) | 64 (50.0%) | 0 (0%) |
| **Surgery** |  |  |  |
| No | 204 (68.2%) | 96 (75.0%) | 2 (5.6%) |
| Yes | 95 (31.8%) | 32 (25.0%) | 34 (94.4%) |
| **Chemotherapy** |  |  |  |
| No/Unknown | 91 (30.4%) | 31 (24.2%) | 1 (2.8%) |
| Yes | 208 (69.6%) | 97 (75.8%) | 35 (97.2%) |
| **Radiation** |  |  |  |
| No | 299 (100%) | 128 (100%) | 36 (100%) |

**Abbreviations:** NR, non-radiotherapy.

**Table S7.** Baseline characteristics of the training and internal-/external- validation datasets in R group

|  | **Training Group** | **Internal-validation Group** | **External-validation Group** |
| --- | --- | --- | --- |
|  | **(N=115)** | **(N=49)** | **(N=20)** |
| **Survival time** |  |  |  |
| Mean (SD) | 63.4 (67.6) | 65.6 (78.2) | 36.4 (26.8) |
| Median [Min, Max] | 29.0 [1.00, 299] | 26.0 [4.00, 383] | 35.3 [11.4, 120] |
| **Age** |  |  |  |
| <40 | 59 (51.3%) | 23 (46.9%) | 9 (45.0%) |
| 40-60 | 42 (36.5%) | 21 (42.9%) | 11 (55.0%) |
| >60 | 14 (12.2%) | 5 (10.2%) | 0 (0%) |
| **Primary Site** |  |  |  |
| Cervix uteri | 87 (75.7%) | 36 (73.5%) | 13 (65.0%) |
| Endo-cervix/Other | 28 (24.3%) | 13 (26.5%) | 7 (35.0%) |
| **Histologic Type** |  |  |  |
| Large | 12 (10.4%) | 5 (10.2%) | 11 (55.0%) |
| Small | 103 (89.6%) | 44 (89.8%) | 9 (45.0%) |
| **FIGO_Stage** |  |  |  |
| I | 48 (41.7%) | 18 (36.7%) | 4 (20.0%) |
| II | 7 (6.1%) | 5 (10.2%) | 8 (40.0%) |
| III | 45 (39.1%) | 20 (40.8%) | 6 (30.0%) |
| IV | 15 (13.0%) | 6 (12.2%) | 2 (10.0%) |
| **N** |  |  |  |
| N0 | 58 (50.4%) | 25 (51.0%) | 14 (70.0%) |
| N1 | 52 (45.2%) | 22 (44.9%) | 6 (30.0%) |
| NX | 5 (4.3%) | 2 (4.1%) | 0 (0%) |
| **M** |  |  |  |
| M0 | 99 (86.1%) | 42 (85.7%) | 19 (95.0%) |
| M1 | 16 (13.9%) | 7 (14.3%) | 1 (5.0%) |
| **Surgery** |  |  |  |
| No | 5 (4.3%) | 4 (8.2%) | 3 (15.0%) |
| Yes | 110 (95.7%) | 45 (91.8%) | 17 (85.0%) |
| **Chemotherapy** |  |  |  |
| No/Unknown | 14 (12.2%) | 4 (8.2%) | 0 (0%) |
| Yes | 101 (87.8%) | 45 (91.8%) | 20 (100%) |
| **Radiation** |  |  |  |
| Yes | 115 (100%) | 49 (100%) | 20 (100%) |

**Abbreviations:** R, radiotherapy.

## 1.3 Supplementary Examples

***Case Analysis***

Case 1: A 50-year-old female patient diagnosed with small cell neuroendocrine carcinoma of the cervix (FIGO stage I) underwent radical surgery. She declined chemotherapy postoperatively. Would it be advisable to recommend adjuvant radiotherapy for this patient after surgery?

A. If radiotherapy were not supplemented, the patient's long-term survival prognosis would be:

|  | Score |
| --- | --- |
| Age | 9 |
| Histology | 0 |
| FIGO-Stage | 2 |
| Node-Status | 0 |
| Therapy | 45 |
| **All** | **56** |


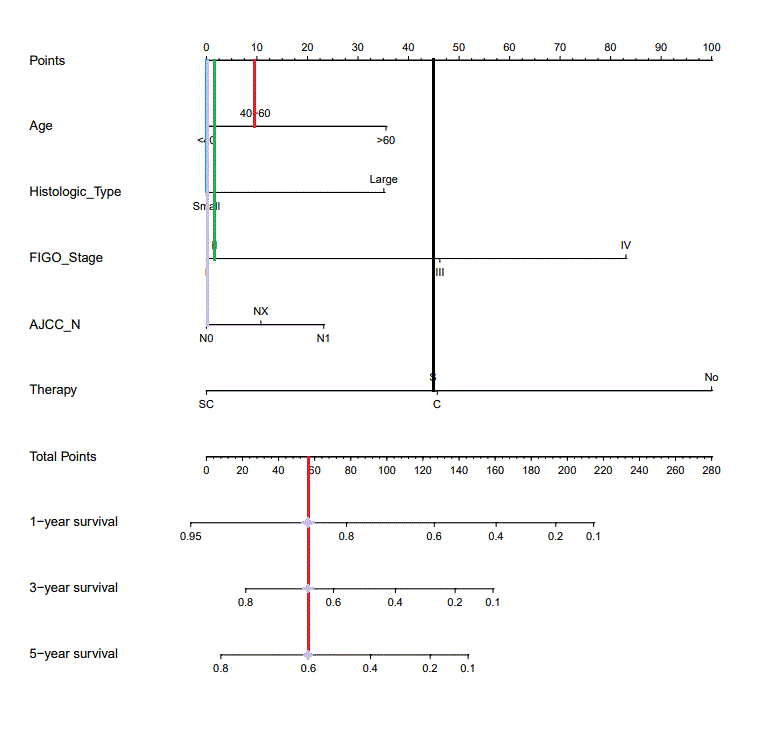


| 1-year survival probability | 3-year survival probability | 5-year survival probability |
| --- | --- | --- |
| ＞80% | 65% | 58% |

B：If radiotherapy were supplemented, the patient's long-term survival prognosis would be:

|  | Score |
| --- | --- |
| Age | 13.5 |
| FIGO-Stage | 0 |
| Therapy | 42 |
| **All** | **55.5** |

**
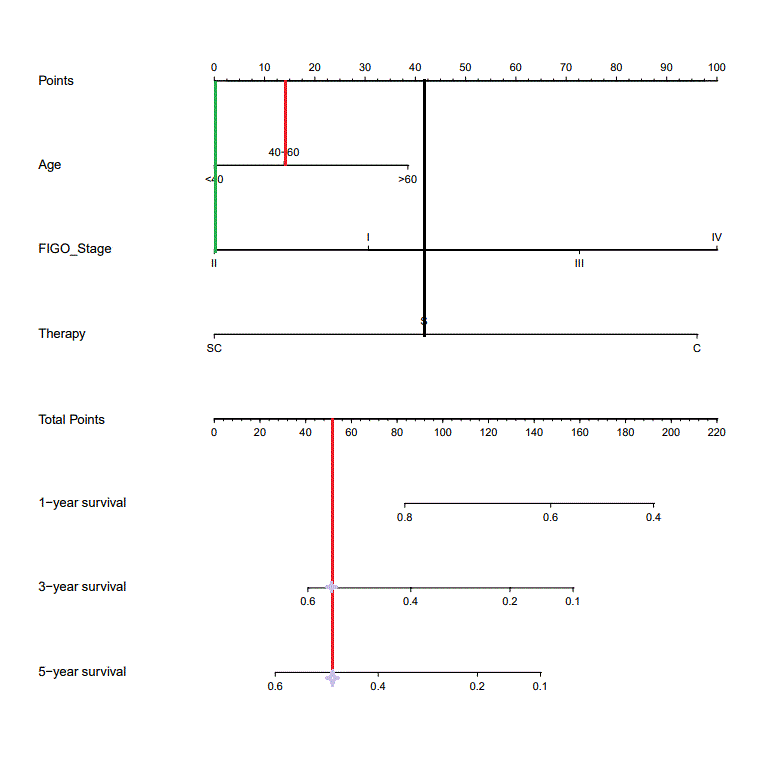
**

| 1-year survival probability | 3-year survival probability | 5-year survival probability |
| --- | --- | --- |
| ＞80% | 55% | 47% |

According to the Nomogram calculation results: The 3- and 5-year survival probabilities of the patient decrease after adjuvant radiotherapy. Therefore, it is not advisable to provide additional radiotherapy.

Case 2: A 47-year-old female patient diagnosed with large cell neuroendocrine carcinoma of the cervix, stage III according to FIGO criteria, with pelvic lymph node metastasis. As there is no surgical indication, we plan to undergo chemotherapy for at least 5 cycles. Is radiotherapy needed during chemotherapy?

A. If radiotherapy were not supplemented, the patient's long-term survival prognosis would be

|  | Score |
| --- | --- |
| Age | 10 |
| Histology | 35.5 |
| FIGO-Stage | 46.5 |
| Node-Status | 23.5 |
| Therapy | 46 |
| **All** | **161.5** |


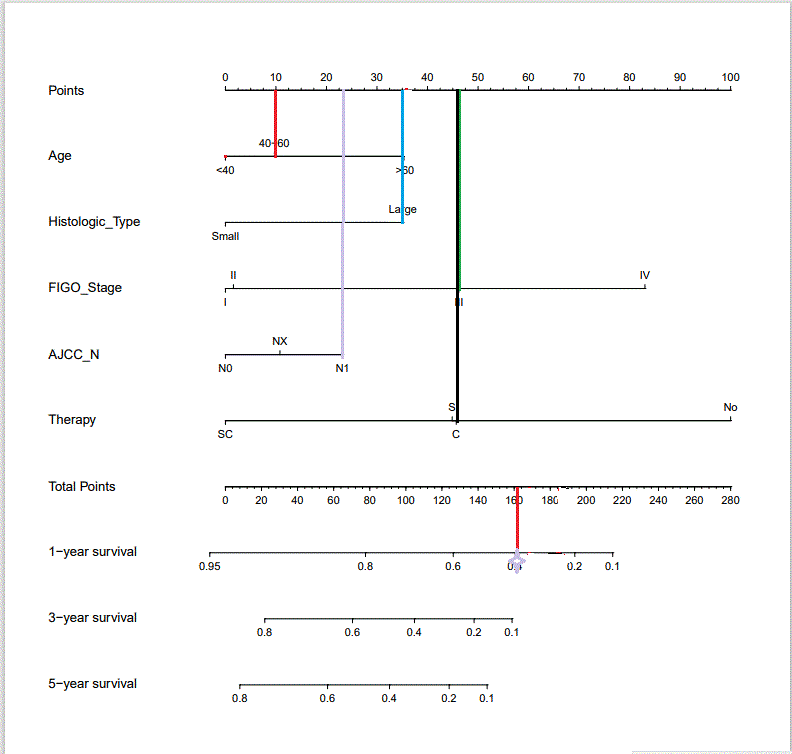


| 1-year survival probability | 3-year survival probability | 5-year survival probability |
| --- | --- | --- |
| 38.8% | ＜10% | ＜10% |

B. If radiotherapy were supplemented, the patient's long-term survival prognosis would be:

|  | Score |
| --- | --- |
| Age | 13.75 |
| FIGO-Stage | 72.5 |
| Therapy | 96.75 |
| **All** | **183** |


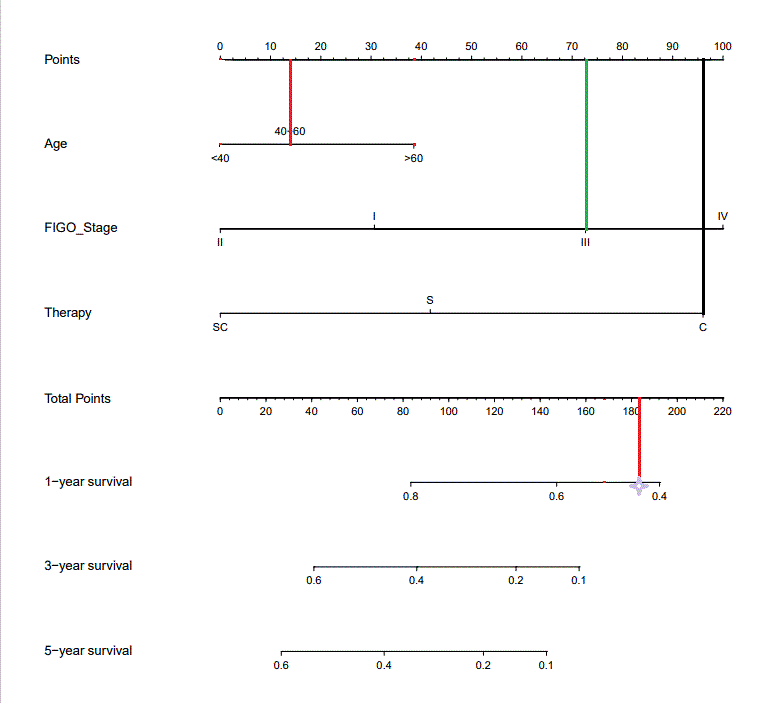


| 1-year survival probability | 3-year survival probability | 5-year survival probability |
| --- | --- | --- |
| 45% | ＜10% | ＜10% |

In this case, the OS for the patient is poor (with both 3- and 5-year survival probabilities being less than 10%). Considering that adjuvant radiotherapy may improve the 1-year survival prognosis to some extent, it may be advisable to consider adding regional radiotherapy at the discretion of the treating physician.
